# Working memory’s pointer system is governed by physical objecthood, not spatiotemporal information

**DOI:** 10.3758/s13423-026-02912-9

**Published:** 2026-04-17

**Authors:** Halely Balaban

**Affiliations:** Department of Education and Psychology, The Open University of Israel, 1 University Rd, Raanana, Israel

**Keywords:** Pointer system, Visual working memory, Resetting, Spatiotemporal information

## Abstract

To properly reflect the dynamic environment, the online representations in visual working memory (VWM) must be constantly accessed and modified when the corresponding items change. Such updating depends on a continuous mapping between each VWM representation and a subset of the external environment, instantiated by a “pointer system.” If this mapping is disrupted, the representations cannot be updated, and instead VWM removes the unmapped representations and starts anew. During this “resetting” process, people are blind to salient changes in the items whose representations are inaccessible, creating a behavioral cost. The goal of this study was to test whether VWM’s pointers are governed by spatiotemporal information or objecthood. In two preregistered studies, participants performed an “online change detection” task, reporting visible changes in items’ shapes, which happened during their (task-irrelevant) movement. Experiment 1 showed that when an intact object abruptly changes its spatiotemporal trajectory, the mapping is sustained, with no resetting cost. Experiment 2 showed that when only half the object changes its trajectory, which effectively splits the object in two, the mapping no longer survives, with diminished performance indicating a resetting process. Critically, a behavioral cost was found even for the half that continued in the same spatiotemporal trajectory, pinpointing objecthood as the driving factor. Together, the results demonstrate that resetting does not reflect mere pointer reassignment, but a specific invalidation of the mapping between representations and the environment. In sustaining this mapping, VWM’s pointer system relies not on spatiotemporal information per se, but on physical objecthood.

## Introduction

As any new driver quickly notices, our environment is hectic. Objects constantly change – in terms of locations, features, and so on – and even steady external stimuli produce varying percepts as a function of viewers’ bodily position or focal point. The key to creating appropriately dynamic mental representations of the world is visual working memory (VWM). This online workspace stores a small amount of relevant visual information in an active state (Luck & Vogel, 2013; Ma et al., 2014). VWM representations can be used by higher cognitive functions, and transformed in real time when items move, change, or interact (Blaser et al., 2000; Drew & Vogel, 2008; Hollingworth & Rasmussen, 2010; Luria & Vogel, 2014).

However, this flexible updating ability requires a way to access the representation of a specific item when the corresponding real-world entity changes (Pylyshyn, 2000). The mechanism responsible for this continuous access is VWM’s *pointer system* (Awh & Vogel, 2025; Balaban & Luria, 2017; Pylyshyn, 2001; Swan & Wyble, 2014; Yu, 2025): a mechanism that establishes a one-to-one mapping between each VWM representation and a specific subset of the environment (mediated by perception).

If VWM’s pointer system is disrupted, VWM representations cannot update, because without a valid mapping they become inaccessible. Instead, a resetting process is triggered (Balaban et al., 2018a, b, 2019, 2023), whereby unmapped representations are removed before new (mapped) ones can be encoded. This happens following a range of mapping-invalidation events, such as when an encoded item is abruptly replaced by another one, making the original item irrelevant. Resetting also happens when a coherently moving object splits into two independent halves, because pre-split there is only one representation, supported by one mapping, and post-split there are two items, none of which matches the original representation.

Resetting is reflected by a transient decrease in VWM load (Balaban & Luria, 2017, 2019; Friedman et al., 2024, 2025; Park et al., 2020), as seen in a lower amplitude of the electrophysiological index of VWM, the contralateral delay activity (CDA; Luria et al., 2016; Vogel & Machizawa, 2004). Furthermore, people struggle to notice changes that occur during the resetting process, presumably because without a valid mapping, VWM representations are inaccessible (Balaban & Luria, 2017; Balaban et al., 2018a, b). When participants watched moving polygons and were asked to indicate whether they changed their shape during the movement, changes that coincided with the split of a coherent polygon were largely missed (note that in sharp contrast to change blindness or inattentional blindness, here the difficulty is in identifying a change in the items participants are actively attending). This damaged performance was highly specific, lacking for changes shortly before or after the split, for items that never split, or when the pre-split halves were clearly marked (e.g., by outlining each half with a thin frame) and thus easily individuated. The neural and behavioral costs associated with resetting form novel tools for studying the VWM-to-environment mapping, by revealing what interrupts its continuity, and consequently, what VWM’s pointer system normally relies on to support the reliable, usable, and flexible nature of VWM representations.

Perhaps the most important question regarding VWM’s pointers is how they are assigned. Most past and present research has argued for a spatiotemporal pointer system (e.g., Thyer et al., 2022), and much of the evidence is in line with this. For example, in multiple object tracking (Holcombe, 2023; Pylyshyn & Storm, 1988) people are able to “hold on” to a pre-defined subset of moving visual items despite being identical to each other and to distractors, and despite constantly changing locations, which could be achieved based on stable trajectories. Moreover, in tracking tasks, people struggle to maintain the identity of items (Horowitz et al., 2007) and even fail to notice changes in the surface features of items (Bahrami, 2003), highlighting the role of spatiotemporal over featural information.

Importantly, in the above-mentioned studies, like in the real world, spatiotemporal information was confounded with objecthood. When resetting-based studies attempted to disentangle these two factors, a different view of VWM’s pointer system emerged, revolving not around spatiotemporal information but physical objects, or “proto-objects” (Pylyshyn, 2001). Two findings are central. First, the same spatiotemporal configuration can lead to either resetting or updating, based on objecthood cues. For example, a colored square splitting into two identical rectangles triggers resetting, while adding thin black frames around each rectangle eliminates resetting and allows the mapping to survive the split (Balaban et al., 2019). This suggests that resetting does not simply reflect spatiotemporal disruption. However, it might be argued that marking each half supports a quick reassignment of pointers, and consequently no resetting occurred.

The second line of evidence comes from a recent EEG study (Balaban et al., 2024) that targeted the intuitive expectations adult humans, as well as pre-verbal infants and non-human animals, hold about physical objects (Baillargeon et al., 1985; Spelke & Kinzler, 2007). Specifically, when people watched simple three-dimensional scenes where objects moved behind an occluder and came back out, if object permanence was violated – by magically removing or adding an object while items are occluded – a resetting process occurred. Critically, if the same events were preceded by the presentation of a “hole” (a black area) behind the occluder, there was no resetting when an object vanished behind the occluder, because the hole explained away the violation (the event was interpreted as an object “falling down”). The events with and without the hole were identical in all spatiotemporal information, so the results suggest VWM’s pointer system is governed by intuitive expectations of physical objects generally, and not only by spatiotemporal continuity. However, this conclusion relies on a specific interpretation of the CDA, which has hardly been used in such complex cases.

In light of this, the present goal was to provide behavioral evidence that VWM’s pointers are not maintained based on spatiotemporal information narrowly, instead marking objecthood as the critical factor. Two preregistered experiments employed the online change-detection paradigm to test for the presence and magnitude of a resetting-cost. Experiment 1 examined whether intact objects that undergo abrupt spatiotemporal changes also incur a cost. In Experiment 2 objects could split, but in a way that enabled comparing different levels of spatiotemporal stability: one half adopted a novel trajectory while the other continued moving in exactly the same way; in both cases an object is destroyed, but only the former further involves spatiotemporal change. If VWM pointers are efficiently reassigned based on spatiotemporal information, the continuing trajectory should not incur a cost. To anticipate the results, abrupt spatiotemporal change was found to be insufficient (Experiment 1) and unnecessary (Experiment 2) for a resetting-cost. This suggests that resetting should be understood as the outcome of a specific invalidation of the (object-based) mapping carried out by VWM’s pointer system.

## Methods

Materials, data, and preregistration protocols are available via the Open Science Framework at: https://osf.io/mwq4r

### Participants

Research was approved by the Open University of Israel Ethics Committee. All participants provided informed consent. Participants were students with normal or corrected-to-normal vision and normal color vision, who received partial course credit.

Sample size was calculated using G*Power (Faul et al., 2007) based on a closely related previous study (Balaban et al., 2018a), aiming for 80% power with ⍺ = 0.05. In Experiment 1, power analysis was calculated based on the cost (i.e., difference between motion conditions) for framed polygons, as this effect was the smallest in the study (to provide enough power to detect spatiotemporal effects that are more subtle than the established resetting effect); with d = 0.8, this required 15 participants. In Experiment 2 (with two groups), power analysis was calculated based on the interaction between Group and Time for the cost, with partial η^2^ = 0.082, which required 20 participants in total, but due to an error, *N* = 30 was preregistered, and so this was the sample size used (divided equally between the two groups).

Participants were replaced if their false alarm rate (i.e., reporting changes when no such changes occurred) was higher than 10%, because this presumably reflects task misunderstanding. This criterion resulted in the replacement of two participants in Experiment 1 (mean FA: 36%; mean FA of remaining participants: 2%) and 15 in Experiment 2 (mean FA: 15%; mean FA of remaining participants: 3%). Including the participants who were removed based on this preregistered criterion did not change the pattern of results; see the Online Supplementary Information for the full results without exclusion. Sample size and exclusion criteria were preregistered. The final sample included 11 females (mean age: 27.7 years) in Experiment 1, and 15 females (mean age: 25.7 years) in Experiment 2.

### Stimuli and procedure

The task was an Online Change Detection paradigm (e.g., Balaban et al., 2018b), used to probe VWM in real time to reveal the dynamics of its pointer system.

Each trial started with a 1,000-ms fixation display (a black cross in the middle of a gray screen), followed by the presentation of a single polygon (1.6° × 1.6° of visual angle from a viewing distance of about 60 cm) at a random location, within a 6° radius around fixation. The polygon was created by randomly combining one right-side half and one left-side half, out of six possible stimuli on each side. Each side of both polygon halves spanned the full potential height, so that each pair of right and left halves could be combined to create one seamless polygon (for a total of 36 possible polygons).

**Fig. 1:**
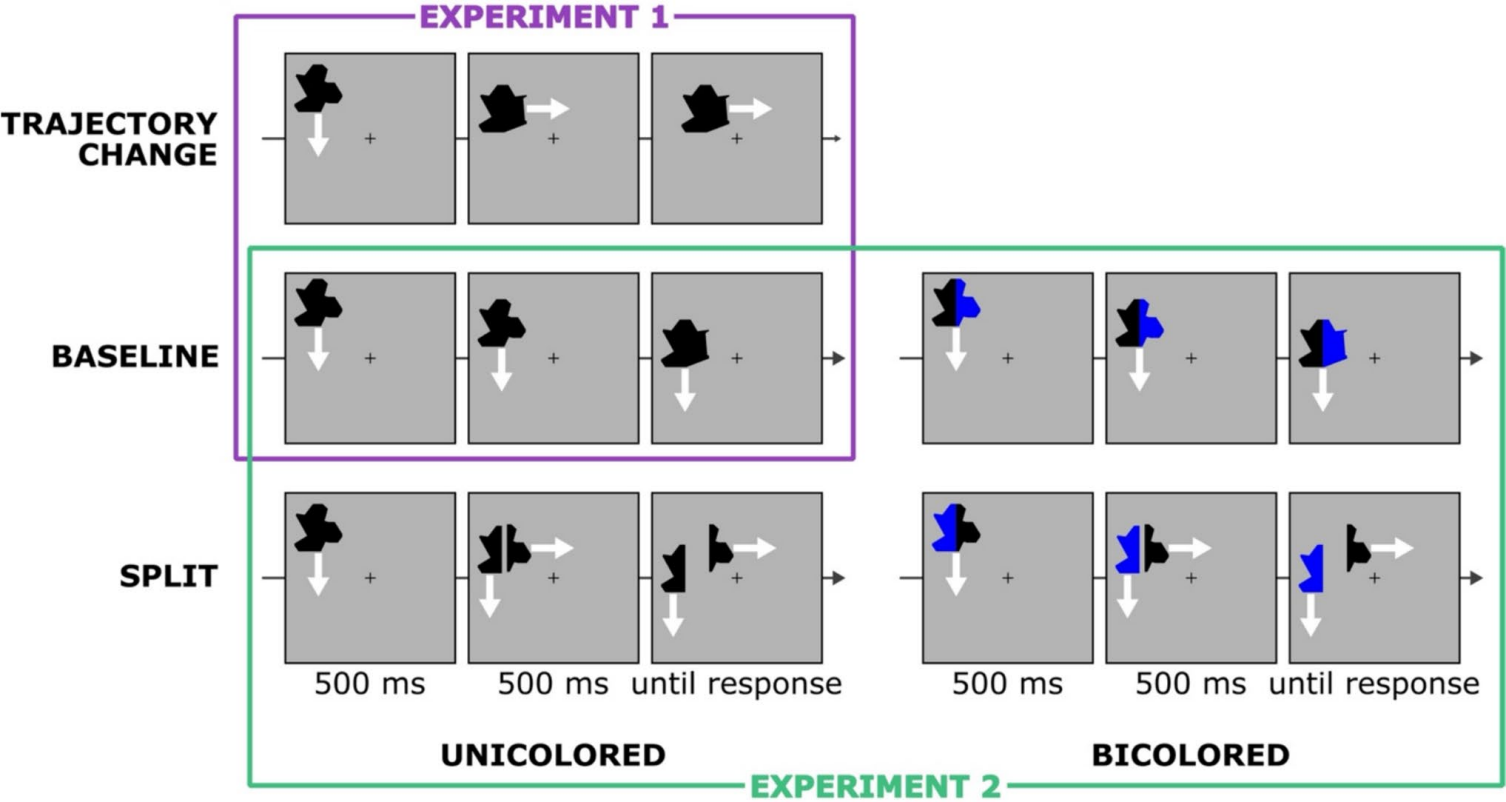
Task and conditions of Experiments 1 (purple frame) and 2 (green frame). Items moved on-screen for 1,000 ms (white arrows indicate the direction of movement and were not visible on-screen) and could change their shape during the movement, which participants were asked to report. In Experiment 1 all items were solid black, and in Experiment 2 one group saw solid black items and the other bicolored items (half black, half blue). The baseline condition in both experiments (middle row) included a random polygon that moved in a constant direction. Experiment 1 additionally included a “trajectory-change” condition, where the polygon abruptly changed direction after 500 ms. Experiment 2 instead included a “split” condition, where only half of the polygon changed direction after 500 ms. Shape changes (50% of trials) were independent of the condition, and could happen after 250, 500, or 750 ms from movement onset (translating to 250 ms before, during, or 250 ms after the potential trajectory change or split)

The polygon moved at a constant speed for 1,000 ms, covering roughly 1.5°. The allowed directions were up, down, left, or right (randomly determined with equal probabilities). There were different possible motion sequences based on condition (see below and Fig. 1). Regardless of the condition, on half of the trials one polygon half changed during the movement to a new half of the same side. These shape changes could happen either 250, 500, or 750 ms from movement onset. Participants’ task was to indicate, in an unspeeded manner, whether there was a shape change or not, using the “D” (no-change) and “K” (change) keyboard buttons. No feedback was provided.

Participants completed 20 practice trials, followed by 480 trials in eight blocks of 60 trials each, with a self-paced break between blocks. The experiment took about 30 min to complete.

#### Experiment 1

There were two movement conditions: Constant, where the polygon continued in the same direction throughout the trial, and Trajectory-Change, where the direction changed by 90° (the new direction was randomly determined out of the two possible directions) after 500 ms. The trajectory change was completely task irrelevant, which participants were explicitly told. In the Trajectory-Change condition, the potential times of shape change translate to 250 ms before, during, or 250 ms after the change in direction. Polygons were uniformly black in both conditions.

#### Experiment 2

There were two groups of participants (15 each, randomly assigned) that differed in the colors of the polygons throughout the task. In the Unicolored group, polygons were all black. In the Bicolored group, polygons had one black half and one blue half (side randomly determined on each trial). Regardless of the color group, there were two movement conditions: Constant (same as in Experiment 1), and Split. The Split condition was similar to the Trajectory-Change condition of Experiment 1, except that now only a single half changed its direction at 500 ms, which effectively splits the polygon in two. In the Split condition, the potential times of shape change translate to 250 ms before, during, or 250 ms after the split. Additionally, the shape change could either happen in the half that continued in the same direction, or in the half that changed direction, with equal probabilities.

### Analysis

The dependent measure was Hit Rate, because the time of shape change can only be defined in trials that included such a change. Results in each experiment were examined via an analysis of variance (ANOVA) with Movement Condition and Change-Time as within-subjects factors, and in Experiment 2, Color-Group was also a between-subjects factor. Additionally, in Experiment 2, changes in the Split condition were broken down relative to the Trajectory-Change: performance for shape changes that happened in the half that changed trajectory was compared to shape changes in the half that continued in the same direction, using an ANOVA with Change-Identity (same/different trajectory half), Change-Time, and Color-Group as factors. Significant interactions with Change-Time are interpreted using planned comparisons at time 0 (control condition relative to the change of direction in Experiment 1, and to the split in Experiment 2), as was done in previous studies (e.g., Balaban et al., 2018b). All tests were two-tailed.

## Results

### Experiment 1: Abrupt trajectory changes for intact objects

In Experiment 1, participants performed a variant of the “online change detection” task, where they monitored for a change in the shape of a moving object, which abruptly switched its trajectory on half the trials. The timing of the task-relevant shape change was manipulated relative to the timing of the irrelevant trajectory change (either 250 ms before/after or concurrently with it), as previous studies have shown that people struggle to notice changes that coincide with events that disrupt VWM’s pointer system (Balaban & Luria, 2017; Balaban et al., 2018a, b).

**Fig. 2:**
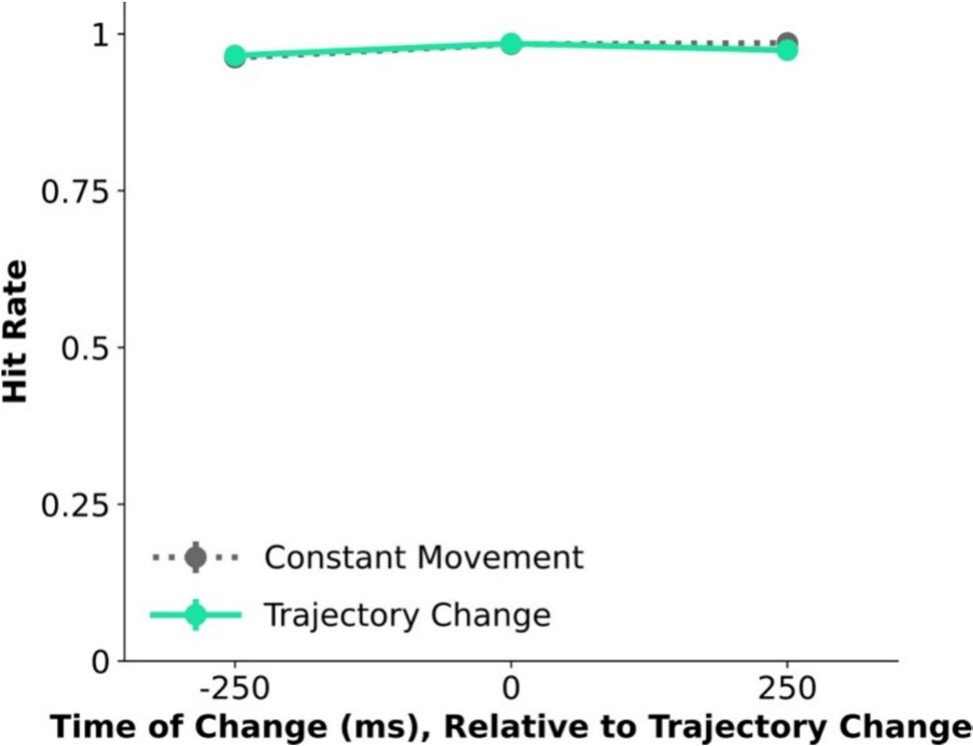
Results of Experiments 1, with abrupt trajectory changes for intact objects. Hit rate (y axis) is plotted for each time of shape change (x axis; defined relative to the potential trajectory change), across the different conditions. Error bars show standard error of the mean

In contrast to previous violations of objecthood, here the sudden spatiotemporal change did not impair the ability to detect changes, as seen in participants’ Hit-Rate (Fig. 2), which was not affected by the Movement Condition (F < 1), and marginally affected by the Change Time (*F*(2,28) = 2.78, *p* = 0.08, partial η^2^ = 0.17), with no significant interaction (*F*(2,28) = 1.18, *p* = 0.32, partial η^2^ =.07). The marginal effect of time was driven by slightly *better* detection for changes that occurred after 500 ms (coinciding with trajectory changes in that condition, but note that there was no interaction) than those that occur shortly before, an effect that was marginally significant (*t*(14) = 2.05, *p* = 0.06, *d’* = 0.53), opposite to the behavioral cost associated with resetting.

The results of Experiment 1 did not reveal any effect of abrupt spatiotemporal changes alone on the ability to constantly track and monitor items. This suggests that the mapping that VWM’s pointer system implements can handle salient spatiotemporal changes and go on supporting VWM updating, meaning that such changes are not sufficient for resetting. The goal of Experiment 2 was to show that spatiotemporal change is also not necessary for resetting.

### Experiment 2: Trajectory changes versus object destruction

In Experiment 2, instead of abruptly changing the trajectory of the entire object, only half of the object changed its trajectory while the other half continued. This manipulation effectively splits the object in two, but in terms of spatiotemporal information, one half maintains its original trajectory. The detection of shape changes was analyzed relative to where the change happened (in the half that also changed direction, or in the one that kept moving in the same direction). Additionally, to estimate the contribution of VWM resetting versus perceptual factors, another group of participants performed the same task but with each half uniquely colored throughout the movement sequence. These bicolored stimuli have been shown to support two pointers even during their joint movement (Balaban et al., 2018a, 2023), meaning that the split should not invalidate the mapping.

**Fig. 3:**
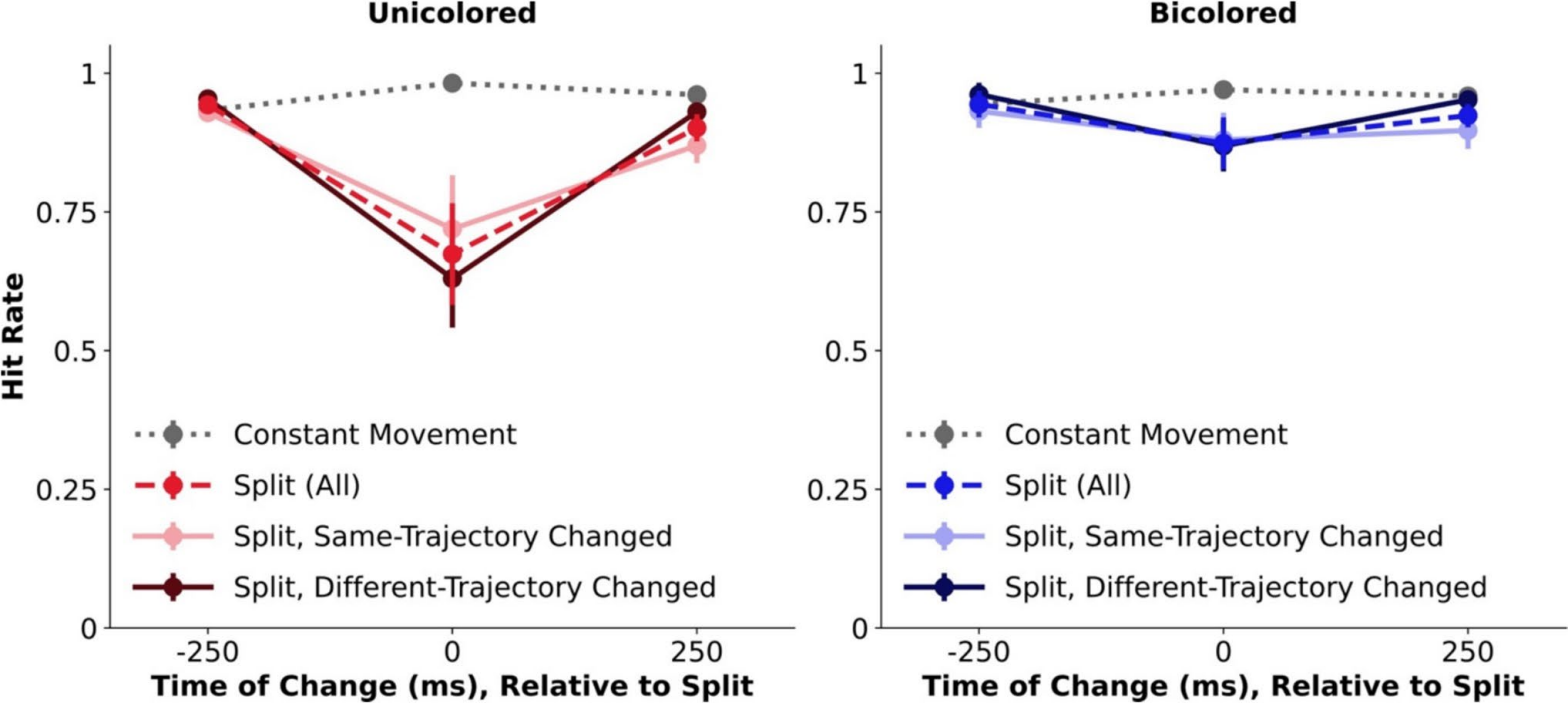
Results of Experiments 2, with abrupt trajectory changes for half an item (i.e., split), in unicolored (left) or bicolored (right) items. Hit rate (y axis) is plotted for each time of shape change (x axis; defined relative to the potential split events), across the different conditions. Split events are shown both by the relationship between the shape change and trajectory change (whether the shape change occurred in the half that also changed trajectory or in the other half), as well as aggregated across this factor. Error bars show standard error of the mean

The results of Experiment 2 (Fig. 3), where only half an item changed trajectory, clearly differed from those of Experiment 1. Aside from effects of Movement Condition (*F*(1,28) = 19.5, *p* < 0.0001, partial η^2^ =.41) and Change-Time (*F*(2,56) = 6.02, *p* = 0.004, partial η^2^ =.18), critically these factors now interacted (*F*(2,56) = 13.61, *p* < 0.0001, partial η^2^ =.33), and also interacted with Color-Group (*F*(2,56) = 4.17, *p* = 0.02, partial η^2^ =.13). Across groups, there was a behavioral cost (lower performance for the Split condition) when changes coincided with the split (Time 0; *t*(29) = 3.85, *p* < 0.0001, *d* = 0.7), and this was also true for each group separately (Unicolored: *t*(14) = 3.35, *p* = 0.005, *d* = 0.87; Bicolored: *t*(14) = 2.58, *p* = 0.02, *d* = 0.67). Replicating previous findings, the cost was more pronounced when both halves had the same color than when each had a different task-irrelevant color (*t*(28) = 2.13, *p* = 0.04, *d* = 0.78). Thus, although the perceptual effect of a split itself decreased the detection of simultaneous changes,^1^ when a coherent object was destroyed there was a large additional cost, presumably due to the pointer system’s mapping being invalidated.

Next, the specific contribution of abrupt spatiotemporal change to the behavioral cost was tested, by comparing shape changes in the half that continued in the same direction to those in the half that changed trajectory. If the problem in detecting changes that coincide with the split reflects the difficulty of reassigning pointers when one spatiotemporal trajectory suddenly transforms to two, it should be found for the half that changed its direction, while performance is expected to be largely intact for the half that continued in the same trajectory. The results showed an interaction of Change-Time and Change-Identity (*F*(2,56) = 11.38, *p* < 0.0001, partial η^2^ =.29). The interaction with Color-Group did not reach significance (*F*(2,56) = 1.9, *p* = 0.16, partial η^2^ =.06), and so the spatiotemporally driven cost is examined across both groups. While changes that coincided with the Split were harder to detect when they happened in the half that changed direction than the one that continued (*t*(29) = 2.7, *p* = 0.01, *d* = 0.49), significant costs were observed for both types of change (in the same-trajectory as well as the changed-trajectory halves; both *t*s > 3, both *p*s < 0.005, both *d*s > 0.6). The Online Supplementary Information reports a reanalysis of a conceptually similar setup (Balaban et al., 2018a) where there was no difference in the size of cost for same- and different-trajectory changes (while the rest of the current pattern was successfully replicated).

The results of Experiment 2 reinforce objecthood as driving the behavioral resetting cost, which is sometimes (though see the Online Supplementary Information) reduced for steady-trajectory items. This spatiotemporal effect, of easier tracking for straight motion, might be strategic^2^ (learning to “hold on” to the continuing half following exposures to splitting events), low-level (i.e., shape changes in pivoting items might be perceptually discounted), or both, which future work could systematically examine. Still, the fundamental effect originates from destroying a coherent object, which in turn disrupts the continuous mapping between VWM representations and the corresponding external items.

## Discussion

The present work tested whether VWM’s resetting process, as a window into its pointer system, can be boiled down to spatiotemporal swerves. Two studies measured the behavioral cost associated with resetting, whereby people struggle to notice changes that coincide with a resetting-triggering event, and differentiated abrupt spatiotemporal changes from objecthood destruction.

Experiment 1 used abrupt trajectory changes in intact items, and found no performance cost for these sudden spatiotemporal transformations when objects were undamaged. In Experiment 2 only half an item changed trajectory, splitting an object in two, which produced a large cost in detecting simultaneous changes. This cost was somewhat larger for changes in the half that changed trajectory, but a non-replication for this specific pattern in an independent sample (see the Online Supplementary Information) suggests that the spatiotemporal effect might not be stable and/or robust. Importantly, even changes in the half that continued in the same trajectory were substantially difficult to notice when they coincided with the destruction of an object. Together, the results show that the generating cause of the resetting cost is not mere difficulty in reassigning pointers based on spatiotemporal information, but instead a true invalidation of VWM’s object-based pointer system.

Notably, the difficulty in noticing salient changes that coincide with resetting is a unique behavioral cost associated with this cognitive process. As also seen here, similar events that allow the mapping to be maintained do not produce a similar blindness, and the present results can therefore be interpreted with regards to VWM’s pointer system specifically. It is also significant to clearly state that VWM’s pointers can be dissociated from the representations that they support: when distinct objects are grouped in VWM, their representations are compressed into fewer VWM-units (consuming less resources), but their pointers remain independent (Balaban et al., 2023; Lando et al., 2025). Thus, explicitly studying VWM pointers as a separate construct from what can be referred to as VWM slots is critical.

The present findings reveal two important consequences regarding the mapping that connects VWM’s representation and the environment. First, the results demonstrate that VWM’s pointer system is governed by objects (Pylyshyn, 2001), and not spatiotemporal information per se (Thyer et al., 2022). The centrality of objects in the VWM-to-environment mapping provides a natural explanation of the wealth of object-based benefits found for the VWM representations that rely on this mapping (e.g., Sone et al., 2021).

It is important to distinguish between the process of object individuation (which is itself beyond the scope here) and its consequences. In determining objecthood, spatiotemporal continuity is central, and in real-world situations it operates alongside other factors, including surface features, Gestalt cues, and semantic knowledge. Then, the products of the individuation process are what VWM’s pointers are assigned to. So, spatiotemporal continuity should be understood as one core aspect of objecthood, which in turn is the critical factor determining VWM pointer allocation and maintenance. This explains how subtle perceptual manipulations, which do not to directly drive pointer maintenance (see Bahrami, 2003; Horowitz et al., 2007), are paradoxically effective in overcoming resetting. Such cues (e.g., changing the color of single object-half) can modify how the environment is segmented into physical objects, producing a downstream qualitative change in pointer assignment.

In a broader context, the results align with a view of VWM and its pointer system as intimately related to physical objects (Balaban & Ullman, 2025; Carey & Xu, 2001). This is supported by recent findings showing that intuitive physical expectations guide pointer-based tracking (Balaban et al., 2024; Lau & Brady, 2020). Spatiotemporal continuity is a necessary-but-insufficient prerequisite for physical objecthood. Violating spatiotemporal continuity violates objecthood as well, but some intuitive physical principles, like rigidity or kind-identity, can be violated while still preserving spatiotemporal continuity, e.g., with abrupt changes in shape. Indeed, at least some such events were already shown to trigger resetting (e.g., Park et al., 2020), and to date every event that was previously found to disrupt the mapping can be reconstrued as a violation of core physical principles (Balaban & Luria, 2025).

A second important aspect of the present findings relates to the resetting process itself. The results suggest that resetting does not involve only “reshuffling,” such that an existing pointer sticks to half of the object and a new pointer is added, because that would imply a cost only for the breakaway half. Rather, resetting appears to consist of a deletion of the original pointer and its complete replacement by new pointers. This interpretation is also supported by the previously reported transient reduction in CDA amplitude, where the lower VWM load could reflect either the removal of unmapped representation or the complete re-encoding of new representations.

A key goal for future research is to delineate the cognitive computations involved in resetting and pointer-based updating. Understanding VWM’s pointer system function, and its interaction with the representational contents of VWM, is highly important due to the role this system plays in everyday dynamic processing. The present work provides an important step in this direction, showing that in VWM, pointers are neither maintained nor invalidated based purely on spatiotemporal information, but on objecthood.

## Data Availability

The datasets generated and/or analyzed during the current study are available via the Open Science Framework at: https://osf.io/mwq4r

## Footnotes

1 The present setup also cannot rule out the possibility that the strong objecthood cue of joint movement encouraged a single mapping to both halves, but see Balaban et al. (2023).

2 The author thanks an anonymous reviewer for suggesting this.

## Declarations

### Open practices statement

The data and materials for all experiments are available via the Open Science Framework at: https://osf.io/mwq4r. Both experiments were preregistered.
